# Molecular data suggest multiple origins and diversification times of freshwater gammarids on the Aegean archipelago

**DOI:** 10.1038/s41598-020-75802-2

**Published:** 2020-11-13

**Authors:** Kamil Hupało, Ioannis Karaouzas, Tomasz Mamos, Michał Grabowski

**Affiliations:** 1 Department of Invertebrate Zoology and Hydrobiology, Faculty of Biology and Environmental Protection, University of Lodz, Banacha 12/16, 90-237 Lodz, Poland; 2 Institute of Marine Biological Resources and Inland Waters, Hellenic Centre for Marine Research, 46.7 km Athens-Sounio Av., 19013 Anavyssos, Greece; 3 Aquatische Ökosystemforschung, Universität Duisburg-Essen, Universitätsstr. 5, 45141 Essen, Germany; 4 Zoological Institute, University of Basel, Vesalgasse 1, 4051 Basel, Switzerland

**Keywords:** Phylogenetics, Biodiversity, Biogeography

## Abstract

Our main aim was to investigate the diversity, origin and biogeographical affiliations of freshwater gammarids inhabiting the Aegean Islands by analysing their mtDNA and nDNA polymorphism, thereby providing the first insight into the phylogeography of the Aegean freshwater gammarid fauna. The study material was collected from Samothraki, Lesbos, Skyros, Evia, Andros, Tinos and Serifos islands as well as from mainland Greece. The DNA extracted was used for amplification of two mitochondrial (COI and 16S) and two nuclear markers (28S and EF1-alpha). The multimarker time-calibrated phylogeny supports multiple origins and different diversification times for the studied taxa. Three of the sampled insular populations most probably represent new, distinct species as supported by all the delimitation methods used in our study. Our results show that the evolution of freshwater taxa is associated with the geological history of the Aegean Basin. The biogeographic affiliations of the studied insular taxa indicate its continental origin, as well as the importance of the land fragmentation and the historical land connections of the islands. Based on the findings, we highlight the importance of studying insular freshwater biota to better understand diversification mechanisms in fresh waters as well as the origin of studied Aegean freshwater taxa.

## Introduction

The Mediterranean islands are considered natural laboratories of evolution, exhibiting high levels of diversity and endemism, making them a vital part of one of the globally most precious biodiversity hotspots and a model system for studies of biogeography and evolution^1–4^. It is estimated that the Mediterranean region, including continental Europe, Northern Africa and Middle East covers approximately 2 million km^2^ which equals ca. 1.3% of the world’s land surface. Yet it is inhabited by ca. 6% of the world’s freshwater species with at least 43% of them considered to be local endemics^5^, however, the freshwater fauna of the Mediterranean islands remains largely understudied.

The Aegean Sea is one of the major sea basins within the Mediterranean, which houses around 7500 islands and islets occurring at a variety of isolation levels and exhibiting different topographic features. The Aegean region is recognized to have, globally, the highest number of islands in a single sea basin^6^. The largest of the Aegean islands is Crete, being also the fifth largest in the Mediterranean region. The majority of the islands were once part of a single landmass called Aegeis, which emerged probably around 23 Ma^7^. In the Middle Miocene, the movement of the African and Eurasian continental plates led eventually to the first fragmentation of Aegeis and to the isolation of Crete from the Peloponnese^3,8^. Further isolations of the land units within the Aegean basin were caused by the formation of Mid-Aegean Trench (around 12–9 Ma), which led to the separation of the central Aegean from the east Aegean islands^3,9^. Another crucial event in the history of the entire region was the closure of the Mediterranean basin that took place at the end of Miocene (6–5 Ma, Messinian Salinity Crisis), leading to complete desiccation and, consequently, to the mass extinction of the marine biota. During that time, many formerly isolated islands regained their connections with the mainland and/or the other islands, which resulted in the exchange of terrestrial and freshwater faunas^3^. Afterwards, some of the connections with the mainland were again temporarily re-established during the Pleistocene, due to eustatic sea-level changes and recurrent glaciation events, with most of the islands gaining their present shape and the current level of isolation at the end of Pleistocene^10^.

Gammarid amphipods are among the most abundant aquatic macroinvertebrates in fresh waters in Europe and, particularly, in the Mediterranean Region^11^. They are known to shape the freshwater macrozoobenthic communities, being recognised as aquatic keystone species^12^. Given the exclusively aquatic life cycle and high level of diversity, freshwater gammarids are considered to be particularly interesting evolutionary models^13^. However, the freshwater gammarid fauna is relatively poorly known with most of the studies on the Mediterranean amphipods being focused on the marine biota. Currently, about 120 freshwater gammarid species have been reported from the Mediterranean region, while only 24 species have been reported from the Mediterranean islands. They belong mainly to two genera: *Gammarus* Fabricius, 1775 and *Echinogammarus* Stebbing, 1899^14–18^. Given that recently a high rate of overlooked diversity was discovered in both the above-mentioned genera [e.g.^19–26^], it may be reasoned that the current number of species already described from the Mediterranean islands is largely underestimated. To date, there have been five species reported from the Aegean Islands with three of them being Cretan endemics (*E. kretensis* Pinkster, 1993, *E. platvoeti* Pinkster, 1993 and the recently described *G. plaitisi* Hupało, Mamos, Wrzesińska & Grabowski, 2018) and with single records of *G. komareki* Schäferna, 1923 from Gökçeada and *G. uludagi* G.S. Karaman, 1975 from Lesbos and Evia^14,15,18,27,28^.

In this paper, we investigate the presence of freshwater populations of *Gammarus* on other Aegean islands. Given the high rate of endemism already observed in numerous organisms from the Aegean archipelago^3^, including freshwater *Gammarus*^14,18^, one can hypothesise that there will be a high level of local endemism on other Aegean islands as well. However, given that final separation of particular Aegean islands occurred at different geological times, with some being isolated since the end of Miocene (e.g. Crete) and some still bearing recurrent land connections with the mainland as late as during Pleistocene (e.g. Evia, Lesbos, Samothraki), it can also be hypothesised that the level of endemism will vary among islands. In this case, the islands that still had land connections in Pleistocene, will perhaps not have endemic lineages, due to possible faunal exchanges with the mainland. These hypotheses are tested by performing DNA polymorphism analyses on four molecular markers: two mitochondrial (COI and 16S rDNA) and two nuclear (28S rDNA and EF1-alpha) in the freshwater populations of gammarids from the Aegean islands and neighbouring, continental regions. By doing so, the aim is to reveal their biogeographic affiliations and the possible origin to provide the first insight into the phylogeography of the Aegean freshwater amphipod fauna.

## Materials and methods

### Sample collection and identification

The study material consisted of 50 individuals of the genus *Gammarus* collected between 2008 and 2018 from 13 sampling sites, including nine sites from freshwater habitats on the Aegean islands, namely Samothraki, Skyros, Evia, Lesbos, Andros, Tinos and Serifos and four sites from mainland Greece (Table 1). Gammarids were sampled using a variety of methods, including collection from gravel, rocks and aquatic vegetation with a hand net or using rectangular kick sample nets (aperture 25 × 25 cm and 0.5 mm mesh size). Samples were sorted on-site, and gammarids were immediately fixed in 96% ethanol. Specimens were examined under a Nikon SMZ 800 stereomicroscope and identified using the available Mediterranean freshwater gammarid taxonomic literature^14–17^.

**Table 1:** Collection sites, MOTU information and GenBank accession numbers for specimens of *Gammarus* used in this study.

MOTU	Site Code	Locality (Island/Mainland Greece; exact location)	Latitude	Longitude	N	GenBank Accession Numbers			
						COI	16S	28S	EF-α
*Gammarus plaitisi*	YGR33	Island; Tinos, Komi	37.6001	25.1333	6	MT999075			
						MT999071			
						MT999058	MT999128	MT999102	MT999090
						MT999085	MT999149		
						MT999049	MT999122		
						MT999084	MT999148		
	YGR37	Island; Tinos, Kardiani	37.5998	25.0667	2	MT999048	MT999121		
						MT999065	MT999134		
	YGR39	Island; Serifos, Kato Dipotama	37.1833	24.4667	5	MT999060	MT999130		
						MT999053	MT999124		
						MT999056			
						MT999080	MT999145		
						MT999041	MT999115	MT999097	MT999087
*Gammarus arduus*	YGR2	Island; Samothraki, Katsabas	40.4961	25.5044	1	MT999064	MT999133		
	GR43	Mainland Greece; Lissos river, near Arisvi	41.0531	25.6183	6	MT999052			MT999089
						MT999050		MT999100	
						MT999043	MT999117		
						MT999066	MT999135	MT999106	
						MT999040	MT999114	MT999096	MW021769
						MT999046		MT999099	
	GR44	Mainland Greece; Apokrimno river, Amfitriti	40.8883	25.9033	4	MT999072	MT999139		
						MT999038			
						MT999082	MT999147		
						MT999074	MT999141		
*Gammarus sp.1*	YGR24	Island; Evia, Stropones	38.6003	23.8908	4	MT999039	MT999113	MT999095	
						MT999044	MT999118		
						MT999077	MT999142	MT999108	MT999093
						MT999047	MT999120		
*Gammarus* sp.*2*	YGR22	Island; Skyros, Loutro spring	38.8329	24.5492	4	MT999081	MT999146	MT999110	
						MT999054	MT999125		
						MT999069	MT999138		
						MT999045	MT999119	MT999098	
*Gammarus* sp.*3*	YGR34	Island; Andros, Ano Menites	37.8167	24.8833	6	MT999068	MT999137		
						MT999073	MT999140		
						MT999061	MT999131	MT999104	MT999091
						MT999083			
						MT999076			
						MT999063			
	YGR36	Island; Andros, Andros city	37.8333	24.9333	3	MT999055	MT999126		
						MT999042	MT999116		
						MT999070			
*Gammarus uludagi*	YGR9	Island; Lesbos, Ampeliko	39.0606	26.3142	3	MT999078	MT999143		
						MT999051	MT999123	MT999101	MT999088
						MT999037	MT999112		
*Gammarus* sp*.4*	GR29	Mainland Greece; Pelion, Chania	39.3935	23.0433	4	MT999079	MT999144	MT999109	MT999094
						MT999059	MT999129	MT999103	
						MT999062	MT999132	MT999105	
						MT999086	MT999150	MT999111	
*Gammarus crenulatus*	GR26	Mainland Greece; Sofades, Sofaditikos river; *locus typicus*	39.3247	22.0934	2	MT999067	MT999136	MT999107	MT999092
						MT999057	MT999127		

### DNA extraction, PCR amplification, sequencing

The DNA was extracted using the standard phenol/chloroform method^29^, implementing the protocol described previously^14^. The extracted DNA was stored at 4 °C until amplification and finally long-term stored at − 20 °C. In the first step, a fragment of the cytochrome *c* oxidase subunit I gene (COI) was amplified using three different primer pairs, depending on the amplification success. In the second step, at least one individual per the delimited Molecular Operational Taxonomic Unit (MOTU) (see below) was amplified for additional markers that were used in phylogeny reconstruction—mitochondrial 16S rRNA and nuclear markers being 28S rRNA and the EF1-alpha gene. All the primer sequences, PCR conditions and original references for all the molecular markers used in this study are provided in Table S1. Afterwards, all PCR products (5 µl) were cleaned up using exonuclease I (ThermoFisher Scientific) and alkaline phosphatase FastAP (ThermoFisher Scientific) according to the manufacturer’s guidelines. Direct sequencing was performed using the same forward primers as for amplification, using the BigDye terminator technology in Macrogen sequencing company. Since the nuclear 28S rRNA marker is over 1100 bp long, it was sequenced both ways, which allowed for obtaining the full coverage, additionally providing better resolution and identification of polymorphic sites. Given that other nuclear marker used in this study, EF1-alpha, has a shorter length and did not exhibit a significant level of polymorphic sites, it was sequenced using only the forward primer.

### Sequence data authentication, editing, alignment, deposition and reference material

All obtained sequences were confirmed as belonging to *Gammarus* via BLASTn searches in GenBank^30^. Subsequently, they were assembled, aligned and trimmed to 625 (COI), 391 (16S), 1107 (28S) and 602 (EF1-alpha) base pairs respectively, using the Geneious 10.0.9 software package^31^. The sequences of two gene-coding markers (COI and EF1-alpha) were translated to check for stop codons. Alignments were performed using the MAFFT plugin with G-INS-i algorithm in Geneious. In case of double peaks and low-quality regions detected in some of the sequences of 28S, the two strands were compared and the dominant signal was chosen at each problematic site. No ambiguous sites were detected in the analysed 28S sequences.

All the sequences were deposited in GenBank (accession numbers to be provided upon acceptance). Additionally, the sequences of all markers used in this study were compiled in the dataset and deposited in the public repository of the Barcode of Life Data Systems (BOLD)^32^, where all the relevant metadata information and sequence trace files are available (10.5883/DS-GAEG).

The dataset was also supplemented by multimarker dataset of 56 reference sequences (30—COI, 10—16S, 11- 28S, 5—EF1-alpha) from the public repositories, representing the related *Gammarus* species from the Aegean islands, mainland Greece and adjacent regions were added to the dataset, along with a single sequence of isopod *Asellus aquaticus* used for rooting the tree (Table S2).

### MOTU delimitation and interspecific relationships

The Molecular Operational Taxonomic Units (MOTUs) were delimited using COI sequence dataset. Firstly, according to the distance-based Automatic Barcode Gap Discovery (ABGD) methodology^33^. The results of the genetic distance-based, ABGD MOTU delimitation were cross-validated with the phylogenetic tree based delimitation methods, namely: Generalized Mixed Yule Coalescent (GMYC) single and multiple models^34,35^ using GMYC Web Server (https://species.h-its.org/gmyc/) and multi-rate Poisson tree processes (mPTP)^36^ using MCMC chain of 50 million iterations with a burn-in of 1 million. Additionally, we have measured patristic distances using Patristic 1.0^37^, according to a patristic distance threshold of 16% proposed for crustaceans at the COI locus^38^, based on the prior observations of morphological differences across multiple crustacean species. We have also measured K2p distances in MEGA7 software to compare them with obtained patristic distances^39^. For tree-based MOTU delimitation methods, we have obtained a consensus tree using BEAST 2.4.7 after performing three MCMC runs of 10 M iterations, sampled every 1000 iterations, using Tamura-Nei model, selected as a best-fit model of evolution with bModel test^40^ and Birth–Death tree model set as a prior, chosen as best-fit tree model using path-sampling. MCMC runs were examined using Tracer v1.7.1 and all the sampled parameters for each studied MOTU achieved sufficient effective sample sizes (ESS > 200). None of the reference sequences mentioned in Table S2 were included in the MOTU delimitation processing.

For final visualisation, the neighbour-joining tree of all COI sequences, using the Kimura 2-parameter (K2p) model of evolution with 1000 bootstrap replicates, was created in MEGA7 software^39^.

### Phylogeny reconstruction, time calibration and history of the diversification

The dataset used for reconstruction of time-calibrated phylogeny consisted of a single representative individual per each delimited MOTU, according to ABGD and patristic distance delimitation methods along with 11 reference *Gammarus* sequences as well as individuals, which represented respective calibration points described below. The substitution saturation was tested in DAMBE 7.0.28^41^, using the index proposed^42^, to assess the potential loss of phylogenetic signal. No significant saturation was detected (Iss value lower than Iss.c value; *p* < 0.01; Table S3) for any molecular marker. Based on the best partitioning scheme selected by the PartitionFinder^43^, we divided the molecular data into seven partitions: 16S, 28S with three codon positions of COI and two partitions of EF1-alpha, one comprising codon positions 1. and 2. and another one with 3. codon position. The time-calibrated phylogeny was reconstructed in BEAST 2.4.7 package^44^, performing three MCMC chains of 100 million iterations, sampled every 2000 iterations, using the best-fit substitution models (all listed in Table S4) determined by bModel test^40^. The optimal molecular clock as well as tree model were chosen via path sampling/stepping-stone procedures using three runs per clock model and tree model, analysing the marginal likelihoods and Bayes factors (BF)^45^, using BEAST 2.4.7. Provided the strict clock was rejected for each partition (BF > 50), we used the uncorrelated log-normal relaxed clock^46^. Given that Yule and Birth–Death tree models were equally supported by path-sampling, we have chosen the Yule model as a tree prior, being a simpler model. Additionally, two more MCMC chains of 100 million iterations, with sampling every 2000 iterations with same substitution models, but with joint COI codon positions and TN93 with G and I set as a consensus best-fit substitution model were performed to determine the estimated COI substitution rate.

For molecular clock calibration, we used five primary calibration points known from literature and related to geological events, as well as one secondary calibration point, which helped to validate the clock calibration based upon the primary calibration points (Table S5). The most recent calibration point is based on the radiation of the endemic *Gammarus* species flock in Lake Ohrid, coinciding with the emergence of the lake itself^47,48^, which most probably took place ca. 2 Ma. The second calibration point is based on the split between the Black Sea and the Caspian Sea populations of *Pontogammarus maeoticus*, estimated at about 4 Ma, connected with the shifts of continental plates, causing the of former Pontian Lake into Black and Caspian Sea, respectively^49,50^. The third calibration point reflects the estimated time of diversification of the *Gammarus fossarum* species complex that took place in the Carpathians, being caused by the Middle Miocene subsidence event between ca. 15 and ca. 17 Ma^19^. The fourth one marks the origin of the Acanthogammaridae family, endemic to Lake Baikal, estimated at 28–30 Ma^51–53^. The oldest calibration point reflects the connection between the Eocene regression of the Paratethys Sea at ca. 37 Ma and the divergence between *Sarothrogammarus* and *Rhipidogammarus* genera^23,27^. The constraints of the calibration points were applied by imposing priors on the respective tree nodes, using lognormal distribution of the MRCA priors, which allowed the incorporation of the possible uncertainty of the data. For cross-validating the ages of the nodes obtained using primary calibration points listed above, we have used four additional calibration points based on the fossil record, using three fossil amphipods and one basal eumalacostracan as an outgroup (Table S6). A detailed description of the fossil calibration points is provided in^54^. Besides using the sequences available for the molecular clock calibration, the reference sequences of *Gammarus* available from the literature were used in the reconstruction of phylogeny to provide further insights into the phylogenetic and biogeographical affinities within the Aegean gammarids (all individuals listed in Table S2).

Parameters of all three runs were examined in Tracer 1.7.1 and reached the ESS values above 200. The runs were combined and resampled with LogCombiner 2.4.7 with 30% burn-in, with the maximum clade credibility chronograms being annotated using TreeAnnotator 2.4.4 and visualised using FigTree 1.4.4^44^.

The history of diversification was inferred using the lineage through time (LTT) plot generated in Tracer 1.7.1 from the 1000 trees, subsampled in Logcombiner, obtained from the Bayesian time-calibrated phylogeny reconstruction performed in BEAST 2.4.7 software.

## Results

### Taxonomic assignment and geographical distribution

All the collected *Gammarus* individuals were morphologically assigned to the so-called *Gammarus pulex* group [sensu^15^] reported from Greece and adjacent areas. Specimens from Andros, Evia and Skyros, were morphologically identified as the pan-European morphospecies *Gammarus pulex* (Linnaeus, 1758). Species from Tinos and Serifos were identified as *Gammarus plaitisi*, previously considered as a Cretan endemic. Specimens from Lesbos and Samothraki were ascribed to *Gammarus uludagi* and *Gammarus arduus* G.S. Karaman, 1975, respectively. The latter has been previously reported from mainland Greece and from the Balkan Peninsula, but never from any of the Aegean islands. *Gammarus arduus* was present in two samples from mainland Greece, namely Arisvi (Thraki) and Amfitriti (Thraki). Material from mainland Greece included samples from the Pelion Peninsula and Sofades (Karditsa, Thessaly) containing other members of *Gammarus pulex* group [sensu^15^] reported from adjacent regions; *Gammarus crenulatus*, described from Greece with its *locus typicus,* being the same as sampled in this study. Since some of the individuals could not be assigned with certainty to any known morphospecies, we have decided to use Open Nomenclature (ON) qualifiers^55^, widely accepted in taxonomic nomenclature. All of those individuals were identified as members of genus *Gammarus.* The individuals from Evia, Skyros and Andros were identified as *Gammarus* aff. *pulex*, fitting some of the diagnostic features of the species with a noticeable variation in setation patterns. Given those uncertainties, they were classified as *Gammarus* sp.1, *Gammarus* sp.2 and *Gammarus* sp.3. The individuals from Pelion were identified as *G.* aff. *birsteini*. However, given that *G. birsteini* is so far known only from limited areas in eastern Turkey and Kazakhstan and that not all of the morphological features fitted the studied specimens, we consider it as a different, yet undiscovered, species, thus assigning it to *Gammarus* sp.4.

### MOTU delimitation, diversity, affiliations and distribution

**Figure 1:**
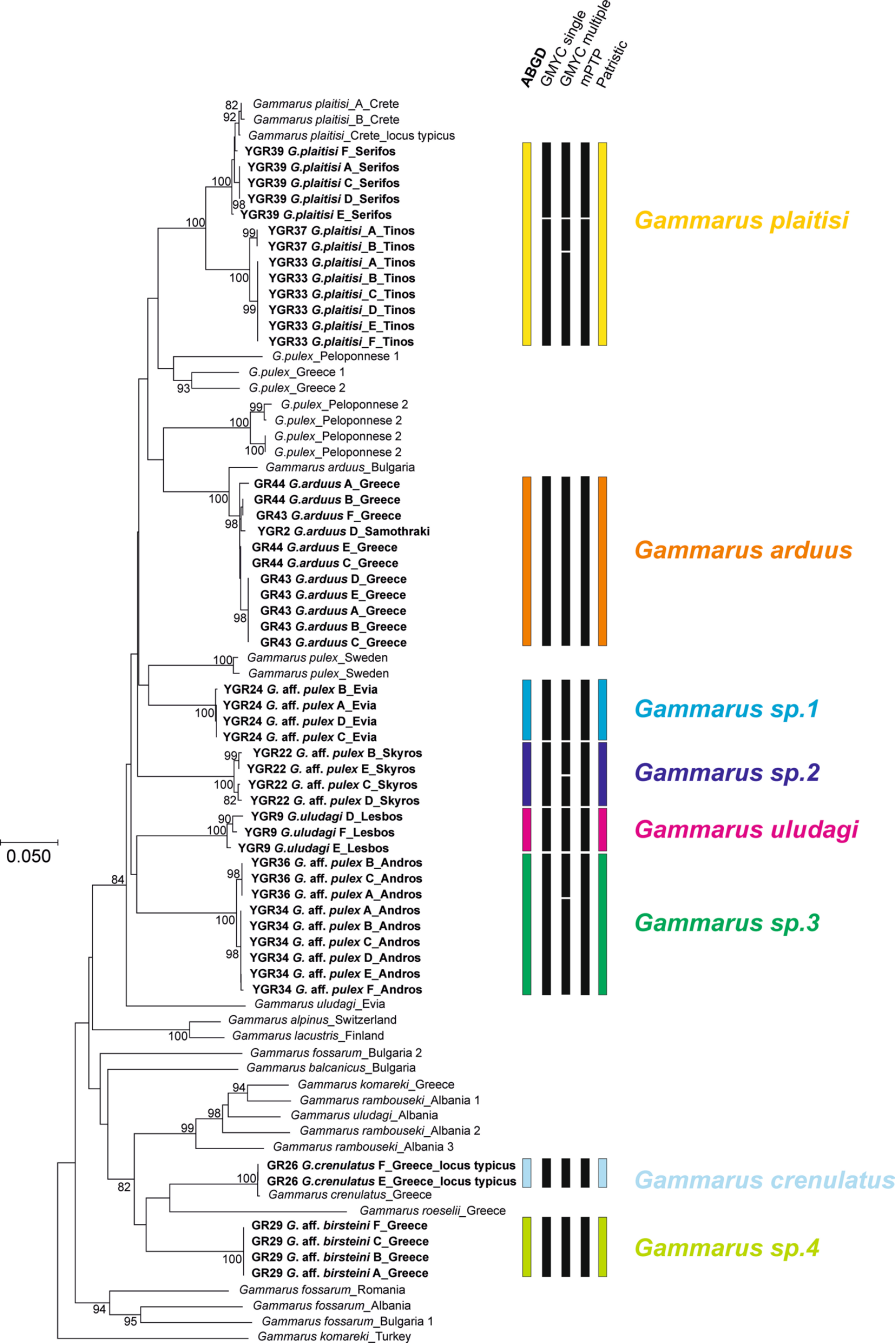
Neighbor-joining COI tree from data obtained in the study (tip labels marked in bold) and mined from NCBI GenBank with the addition of the outgroups. The numbers by respective nodes indicate bootstrap values ≥ 0.75. The scale bar corresponds to the K2p distance. The bars represent different delimitation methods used; colours used for ABGD MOTUs correspond to those presented in other figures.

The ABGD, along with MOTU delimitation based on the patristic distance, supported the existence of eight distinct lineages of *Gammarus* present in our material collected from the islands and from mainland Greece (Fig. 1). For tree-based delimitation methods, the single species hypothesis was rejected for both single and multiple approaches (result of Likelihood Ratio tests < 0.0001). The delimitation results slightly differed among the methods used, namely GMYC single threshold model, mPTP and GMYC multiple threshold. The first two methods indicated the presence of ten MOTUs, with two extra lineages within *G. plaitisi* and *G. arduus*, respectively. Moreover, GMYC multiple threshold model supported twelve MOTUs, splitting *G. plaitisi* into three units, as well as two extra lineages within *Gammarus* from Skyros and Andros. We have chosen both ABGD, cross-validated with patristic distance method as the main delimitation methods due to its most conservative approaches (Fig. 1). Moreover, the patristic distances seemed to be also congruent with the K2p distances, which further supported the delimited MOTUs (Table S8). All of the insular MOTUs grouped together with other *Gammarus pulex* sequences, accompanied by other members of the *Gammarus pulex* species group including *G. uludagi*, *G. lacustris* or *G. alpinus* (sensu Karaman & Pinkster, 1977a). However, the samples from mainland Greece with *G*. *crenulatus* and *G*. sp.4 grouped, with high bootstrap value, together with a reference sequence of *G*. *roeselii*. The latter belongs to the so-called *Gammarus roeselii* group [sensu^16^ (Fig. 1).

**Figure 2:**
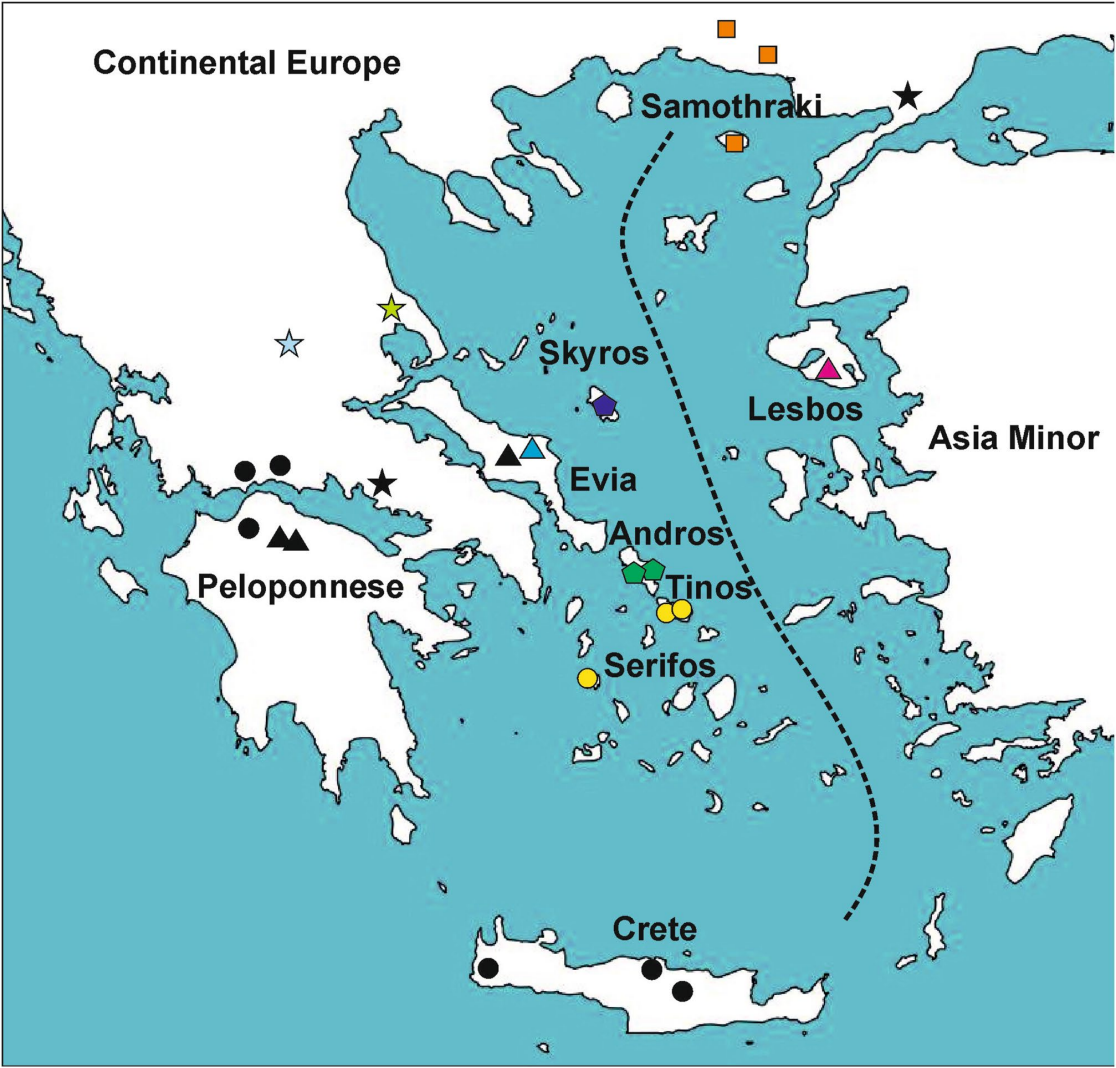
Map of the sampling sites on Aegean islands. The colours and symbols correspond to those presented in other figures. The map was generated using QGIS 2.18.3^81^ (https://www.qgis.org/en/site/).

Each of the studied islands is presumably inhabited by a single MOTU only, with the exception of Evia, where both MOTUs of *Gammarus* sp.1 from this study and *G. uludagi* are present (Fig. 2). In most cases, a MOTU found on one island is present neither on any other island nor on mainland Greece. The only exception is *Gammarus plaitisi* present on Tinos and Serifos as well as on Crete^14^ and *Gammarus arduus* present on both Samothraki and on the mainland (Fig. 2).

### Phylogeny reconstruction, molecular dating and history of the diversification

The estimated COI substitution rate reached the value of 0.0118 ± 0.00677 substitutions/site/My, which is congruent with the substitution rates reported in other studies dating divergences in other crustaceans and arthropods, including freshwater amphipods [0.007–0.0177 substitutions/site/My]; [e.g.^25,54,56–58^].

**Figure 3:**
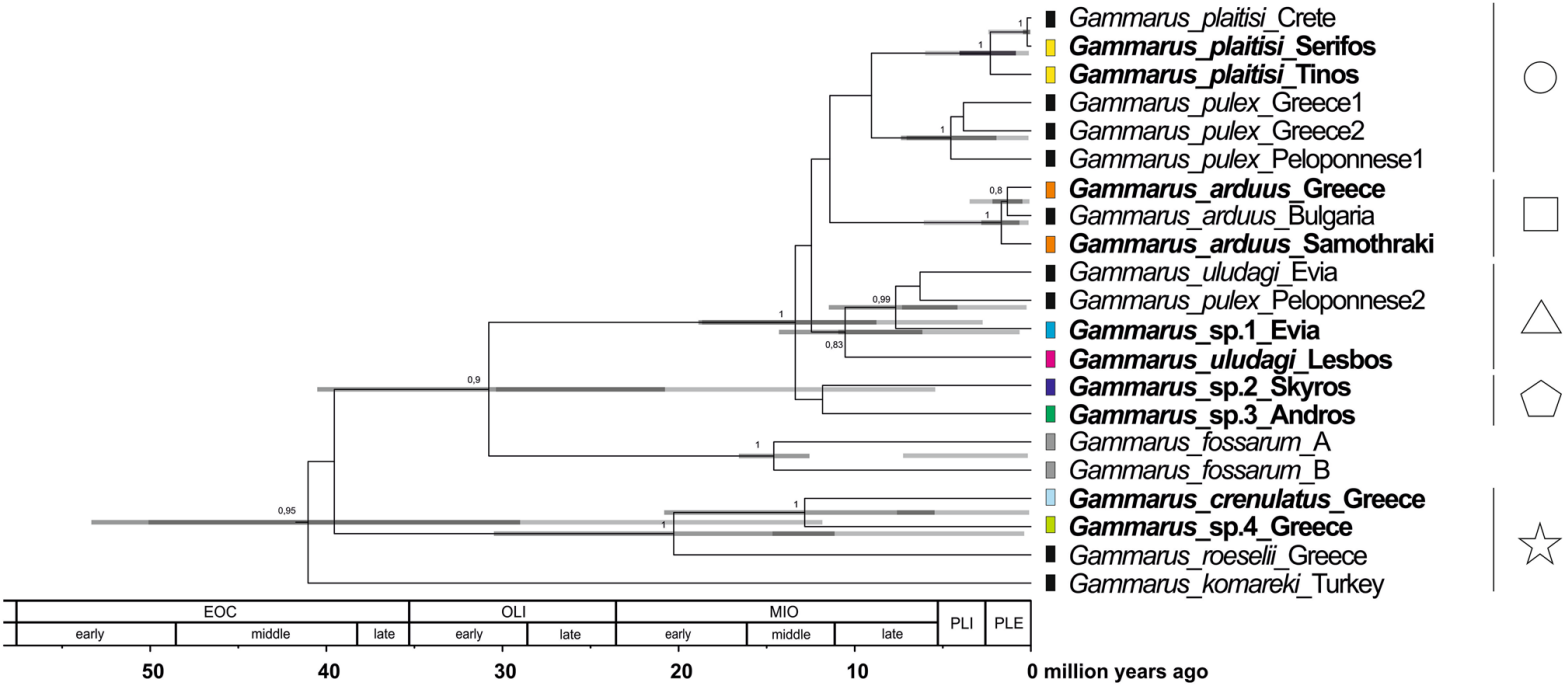
Maximum clade credibility, time-calibrated Bayesian reconstruction of phylogeny of Aegean Gammarus MOTUs. Phylogeny was inferred from sequences of the mitochondrial: COI, 16S rRNA markers and nuclear: 28S rRNA,EF1-α markers. The numbers by respective nodes indicate Bayesian posterior probability values ≥ 0.8. The coloured bars represent ABGD delimitation method, with colours, and symbols and shapes corresponding to those presented in other figures. Dark grey node bars represent 95% HPD obtained from the primary calibration points, whereas the light grey node bars represent 95% HPD obtained from the fossil calibration points. EOC—Eocene, OLI—Oligocene, MIO—Miocene, PLI—Pliocene, PLE—Pleistocene.

The ages of the nodes obtained using primary calibration points were generally congruent with the dating of the nodes through fossil calibration, however the 95% HPD values were generally wider than those obtained through primary calibration scheme (Fig. 3, Table S7). It was expected as the calibration points used in fossil calibration scheme derive from outgroups more distant to the target taxa used in this study and the dates have considerable uncertainty. Since the topology of the obtained consensus tree is congruent (Fig. S1), and all the well-supported nodes were retrieved as well as their dates see

m to be supported by both calibration schemes, we have decided to present and discuss the nodes obtained via primary calibration scheme as reliable proxy for species’ divergence.

The time-calibrated phylogeny revealed that divergence within the *Gammarus pulex* group from the studied region started around 12 Ma (95% HPD: 18.9—8.8 Ma) (Fig. 3). All of the deeper divergence events between the insular taxa seem to take place between 12 and 8 million years ago. At that time, *G. platisi* seemed to diverge from the *G*. *pulex* lineage from mainland Greece. *Gammarus uludagi* from Lesbos diverged from its conspecific from Evia as well as *G*. *pulex* from Evia and Peloponnese and *G*. *pulex* from Skyros and Andros diverged from other lineages (Fig. 3). During the same timescale, about 11.5 Ma (95% HPD: 20.8–5.5 Ma), *G*. *crenulatus* diverged from *G*. sp.4. These two species, along with *G*. *roeselii*, could have separated from *Gammarus pulex* group members already in the Eocene, around 40 Ma. More recently, in the Pliocene and Pleistocene, diversification events probably took place only in the insular populations of *G. plaitisi*, about 2.5 Ma (95% HPD: 4.1–0.9 Ma) as well as in *G. arduus*, where the insular population from Samothraki diverged from the mainland conspecifics, most probably about 2 Ma (95% HPD: 2.8–0.7 Ma). The youngest divergence seemed to take place in late Pleistocene, about 0.2 Ma (95% HPD: 0.45–0.03 Ma), when the population of *G. plaitisi* from Serifos apparently diverged from the one from Crete (Fig. 3).

The lineages-through-time plot (Fig. S3) shows that the accumulation of lineages remained rather constant over time, with no significant increase in lineage accumulation.

## Discussion

### Diversity and distribution of Aegean insular freshwater fauna

In this study, we provide the first evidence of the presence of freshwater populations of *Gammarus* on five Aegean islands, namely Samothraki, Skyros, Andros, Tinos and Serifos, with three of these populations most probably representing the new, distinct species supported by all delimitation methods used in this study. The same holds true for another, possibly new species, inhabiting the limited area in mainland Greece. The ABGD approach used in this study as the main MOTU delimitation method is considered to be more conservative compared to tree-based methods like GMYC and closer to the species distinction provided by taxonomists [e.g.^33,59^]. In some cases, these methods over-split putative entities, depending on the overall genetic distances differential or sampling bias^60,61^. However, cross-validating those methods with the conservative approach of patristic distance raises up the probability of the congruence of delimited taxa^38^. On the other hand, it still poses the question of unanimity of the phylogenetically delimited species with the biological species concept^62^. Recently, based on the experimental observations done on freshwater gammarids, it was argued that lineages separated by a genetic distance exceeding 4% are less likely to form precopulatory pairs and thus, might be reproductively isolated^63^. Even though amphipods diverged by ca. 16% still formed precopulatory pairs under laboratory conditions, this was never observed in the field. However, no further evidence supporting the cross-lineage fertility and presence of hybrid offspring produced in the laboratory conditions was provided in referenced study. It was then argued by the authors that lineages separated by ca. 16% living in sympatry seem to exhibit prezygotic barriers, preventing them from mating with divergent counterparts. In this case, the majority of the insular Aegean species were isolated by more than 16% genetic distance from the closest related lineage, both when using commonly applied for calculating genetic distances Kimura-2-parameter model (K2p) as well as the patristic distance (comparison in Table S8). According to the 4.3% K2p genetic distance threshold proposed for gammarids by^64^ and even more conservative, 16% patristic distance threshold proposed by^38^, one may suppose that they are likely to represent separate and reproductively isolated lineages.

Up to now, in the Aegean basin, members of the genus *Gammarus* were reported only from Lesbos, Gökçeada (Imbros), Thasos, Evia and Crete^15,27,28,65^. Interestingly, none of these records indicated any endemic insular species. Our results reveal that almost every lineage is endemic to one island only (Fig. 2). Apart from the recent study by^14^, describing the new species *G. plaitisi* and *G. uludagi* from Evia provided by^27^, there were no other molecular studies conducted on any Aegean gammarids. Given that molecular studies on freshwater insular gammarids are scarce, one could expect a high number of overlooked diversity on the islands. Considering the fact that Aegean archipelago is characterized by an exceptionally high level of endemism confirmed in numerous biota^3^, it is also probable that some of the overlooked Aegean lineages may, in fact, represent undiscovered endemic taxa. That is a particularly valid assumption for the distinct lineages from Evia, Skyros and Andros, as no individuals belonging to these MOTUs have been reported from elsewhere nor detected in our study.

### Miocene diversification of the Aegean freshwater insular gammarids

**Figure 4:**
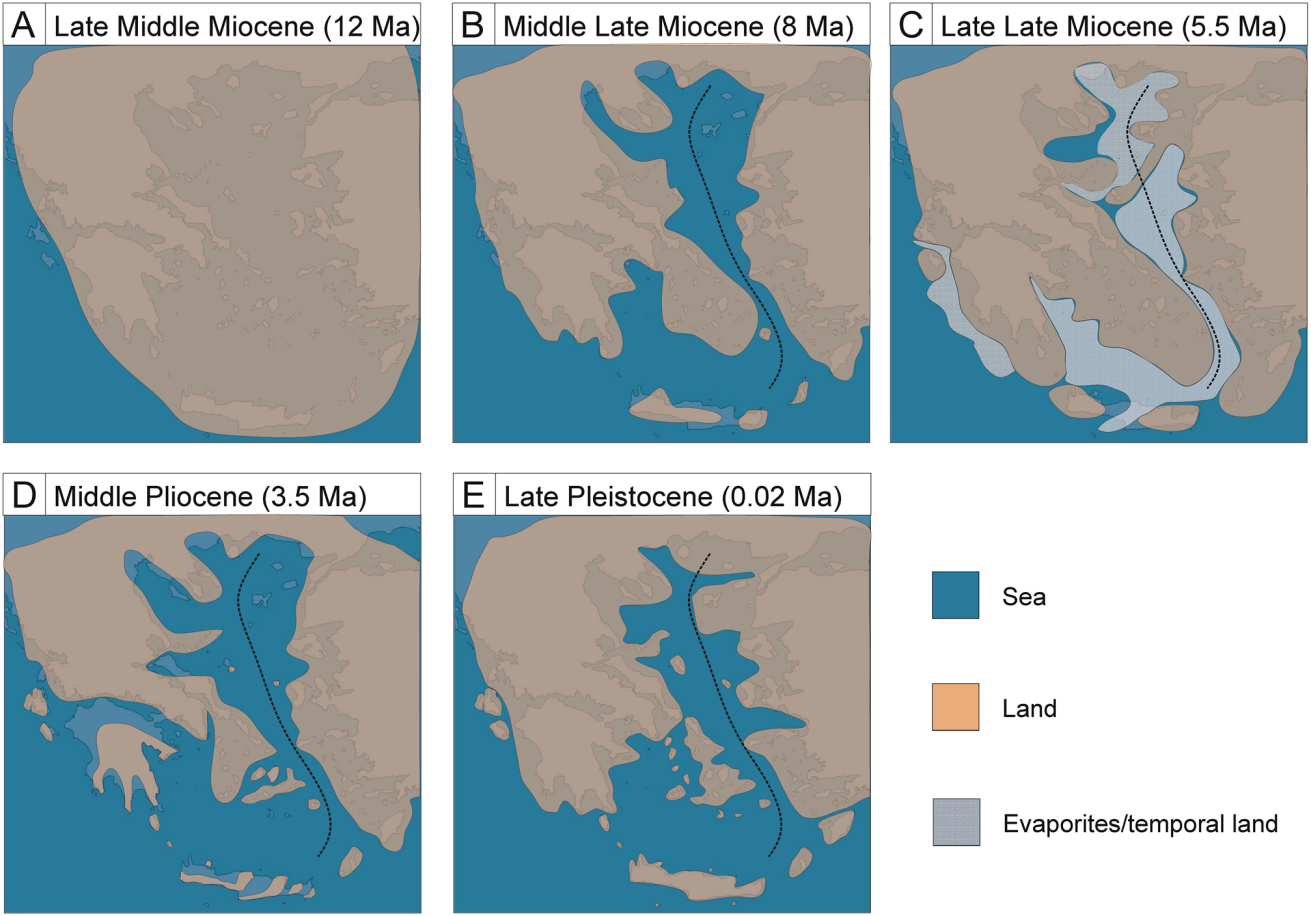
The selection of paleogeographical maps of the Aegean Region, including its islands (after Popov et al.^82^). The dotted line represents the approximate location of the Mid-Aegean Trench. The maps were generated using QGIS 2.18.3^81^ (https://www.qgis.org/en/site/).

Our results suggest that diversification of freshwater gammarids on the Aegean islands started in the Middle Miocene, around 12 Ma (Fig. 3). Up to that point, the Aegean region remained a single landmass (Aegeis), comprising not only all the present islands but also the Balkan Peninsula and Asia Minor^7^. Around 12 Ma, the fragmentation of the Aegeis landmass began, due to collision of the African tectonic plate with the Eurasian plate in the Middle Miocene^66^. In fact, the movements of landmasses during that time supposedly induced divergence events in numerous freshwater crustaceans including amphipods, both epigean^20,21,25^ and subterranean^67^, isopods^68^ and crabs^69^. Moreover, at that time, the formation of the Mid-Aegean Trench started and was fully accomplished by 10–9 Ma, resulting in separation of the western part of the Aegean region from the eastern one (Fig. 4)^9^. These events have led to numerous isolation and diversification episodes in the Aegean fauna, which are reflected in modern distribution patterns^3^. Interestingly, in the Aegean, Middle Miocene events are, in general, known to affect the divergence mainly of terrestrial taxa, such as snails, beetles, isopods and scorpions^3^. Seemingly, all of the Aegean freshwater biota diverging after the end of Miocene or more recently, in Pliocene and/or Pleistocene including species of crabs, crayfish and snails, used temporarily existing land connections^70–72^. Our results suggest, however, that the divergence of the Aegean insular freshwater gammarids was most probably affected by the Middle Miocene land movements and fragmentation, which also might have had a significant impact on their current distribution. For example, the divergence of *G*. *uludagi* from Lesbos, belonging to the eastern Aegean islands, from its conspecific from Evia, coincides with the approximate time of the formation of the Mid-Aegean Trench. The same is true for the divergence of *G*. *pulex* inhabiting the same island from the lineage inhabiting Peloponnese, being on the western side of Aegean basin. This supports the possible connectivity of the divergence with the geological history. It is also suggested that the formation of the Mid-Aegean Trench might have played a role in the evolution and dispersal of freshwater *Pseudorientalia* snails^73^. The authors suggest that limited distribution of *Pseudorientalia,* currently inhabiting only Aegean islands east of the Mid-Aegean Trench, might be associated with the formation of this biogeographical barrier and subsequent regional land fragmentation in the Middle Miocene. Similarly, the land fragmentation in the Middle Miocene most likely affected other divergence events shown in our data, such as the divergence of *G. plaitisi* from Crete, Tinos and Serifos from *G*. *pulex* inhabiting mainland Greece, including the Peloponnese, which coincides with the isolation of Crete and the separation on the islands from Peloponnese (Figs. 3, 3). It also supports the results of the previous study on the divergence of Cretan *G. plaitisi* (Hupało et al.^14^). The land separation at that time most probably resulted also in the divergence between the *Gammarus* from Andros and the one from Skyros (Figs. 3, 4). However, given the low posterior probability of that node, still more data is needed to determine the actual relationships between these taxa.

### Most recent Plio–Pleistocene diversification events

Although major divergence events in Aegean freshwater gammarids took place in the Middle Miocene, some of them occurred more recently, in the Plio-Pleistocene (Fig. 3). The Pliocene water level fluctuations, as well as Pleistocene glaciation events with associated sea regression and climate aridification, strongly affected the river systems and promoted the diversification of numerous biota in the Mediterranean Region^3,21,74^. Several studies have confirmed that major European peninsulas, including the Balkan Peninsula, served as refugia and diversification hotspots for numerous taxa, eventually becoming starting points for their dispersal^21,25,75^. In the Aegean, this was also the time of intense diversification events for numerous vertebrate taxa, mostly involving ‘herptiles’ and mammals^3^ and references therein. Fossil evidence indicates the presence of two pygmy elephant species in the Aegean region, which diversified in the Pleistocene and Holocene^3^. Our data suggest that the separation between *G. arduus* from Samothraki and the mainland conspecifics took place during the same time, from the late Pliocene to the beginning of the Pleistocene (Fig. 3). This makes Samothraki, based on the up-to-date molecular evidence, the only known Aegean island not inhabited by gammarid endemics (Fig. 1). Favourably, this divergence event could be associated with the recurrent Pleistocene land connections between the island and the continent^3,10^. One could argue then for the plausibility of a similar scenario for the other confirmed records of gammarid presence in the Aegean, given that islands like Lesbos, Gökçeada and Thasos—all sharing the temporal land connections with the continent at a similar time as Samothraki did^10^. However, up to now, no molecular evidence is available to confirm or reject such a hypothesis. On the other hand, although Evia was connected with the mainland in Pleistocene, the lineage most likely diverged earlier, in Miocene (Fig. 3). However, this might also be due to undersampling from the mainland and neighbouring islands.

Even more recent is the diversification within *G*. *plaitisi* from the three Aegean islands: Crete, Tinos and Serifos (Figs. 2, 3). By confirming the presence of *G. plaitisi* on Tinos and Serifos, we are rejecting the earlier-proposed alleged endemism of this species on Crete^14^. This finding, along with the very low intraspecific haplotypic diversity on Crete, confirms Plio/Pleistocene dispersal of this species suggested by the authors^14^. Still, it is unclear how the species dispersed from Serifos to Crete, as there were no known temporal land connections between these two islands in Pleistocene (Fig. 4)^3^. It is equally puzzling to the evolutionary history and dispersal of the freshwater *Potamon* crabs, that diverged and dispersed to Crete, as well as, to Cyprus during Pleistocene, where no land connections were known to exist between the islands and the mainland^70^. It is suggested by the authors that early humans might have aided in the dispersal of certain taxa, including crabs, which would then mean that the arrival of these freshwater biotas to Crete may be very recent. Another plausible scenario involves the passive dispersal by birds [e.g.^76^], already suggested for freshwater *Daphniola* snails inhabiting distant eastern Aegean islands^77^. It is argued that the snail lineages probably diverged recently, in Pleistocene, where no land connection was presumably present between those islands, and thus, the dispersal was possible either due to the mediating factor or through the successive dispersal through neighbouring islands^77^. It might be the case also for *G. plaitisi* with possible intermediate, yet still undiscovered, populations, e.g. on Milos or Kythera and Antikythera islands.

### Taxonomic affiliations of the Aegean Gammarus

The results of our study provide new evidence for rejecting the monophyly of *Gammarus pulex* with three potentially new distinct taxa within this morphospecies (Fig. 3). These findings confirm the recently observed high cryptic diversity and lack of monophyly in numerous widespread European freshwater gammarids [e.g.^19–22,26,78,79^]. Interestingly, our data also support the polyphyly of *Gammarus uludagi*. This finding questions the reliability of the specimen recorded from Evia, as the species was originally described and reported from the western part of Asia Minor and the island of Lesbos^15^. Nevertheless, the incongruences in the species’ integrity raise further questions about the taxonomic congruence of other formerly described species (Fig. 3). This seems to be especially valid for specimens assigned to *Gammarus pulex*. The species exhibits a significant level of intraspecific morphological variation in several characters such as e.g. the number of segments in flagella, the shape of epimeral plates, the number of spines and the setation pattern on various body parts^15^. In our study, we have observed mostly the differences in setation on epimers, pereiopods and uropod 3. Although we did not recognize any stable patterns which could reliably distinguish the specimens from particular lineages, we believe that a detailed morphometric study could help to resolve the taxa boundaries. Moreover, the taxonomic affiliation of *G. crenulatus* shown in our results (Figs. 1, 3) also suggests the incongruence of morphogroups, the so-called *Gammarus pulex*, *Gammarus roeselii* and *Gammarus balcanicus* groups, formerly described by^15–17^. Even though *G. crenulatus* was originally assigned to the *Gammarus pulex* group, our results suggest that it is, in fact, more closely related to *G. roeselii* than to *G. pulex* (Fig. 3)*.* Most of the recent thorough phylogenetic studies on gammarids^23,27^ did not include any molecular data on *G. crenulatus*, so more studies are needed to fully resolve this matter. The divergence between *G. roeselii* from mainland Greece, *G. crenulatus* and *G*. sp.4 took place around 20 Ma, well before the fragmentation of Aegeis. It suggests that other processes might have played a role in their divergence, however again more data is needed to reveal the detailed evolutionary history of this group. These data, along with other recent findings^21^ support the need for a comprehensive revision of *Gammarus pulex* and further studies on *Gammarus roeselii* morphospecies, incorporating an integrative approach using the detailed morphological information, ideally, combined with extensive molecular data. Using such a perspective would provide a major step towards fully resolving the species’ phylogenetic relationships.

## Conclusions

The results of our study suggest a high level of local lineage endemism for gammarids in the Aegean islands, which is in agreement with previous studies showing that several taxonomic groups exhibit a high level of local endemism in this part of the Mediterranean. . The presence of *G. arduus* on Samothraki supports our hypothesis that the level of endemism will vary between the islands, with the absence of endemic lineages on islands that were still connected with the mainland during the Pleistocene. On the other hand, the presence of distinct, separate MOTUs on Evia and Lesbos leads to reject this hypothesis; however, more data are needed both from the regions adjacent to Evia as well as from inland waters of Turkey, where *G. uludagi* has been reported. Moreover, the results of our study support the presence of at least four yet undescribed gammarid species, three of which are endemic to respective Aegean islands they inhabit, namely Evia, Skyros and Andros.

The results of the time-calibrated phylogeny indicate multiple origins and different timescales of differentiation for the Aegean insular freshwater gammarids. The biogeographic affiliations of the studied insular taxa indicate their continental origin as well as the importance of the land fragmentation and the historical land connections of the islands that most probably influenced the evolutionary history of the Aegean biota. Moreover, the deep divergences inferred from the reconstructed phylogenies indicate that most gammarid species present in the Aegean islands diverged well before the final isolation of some islands, indicating that they may be considered the only known freshwater taxa belonging to ‘the old colonizers’ (sensu^3^). These results not only provide important evidence supporting the survival of at least some freshwater taxa of the Messinian Salinity Crisis, but also may be highly useful as calibration points e.g. formation of Mid-Aegean Trench or recent isolation of Samothraki from the mainland, for future studies of Aegean taxa.

Given the scarcity of available data, one cannot exclude further discoveries of freshwater gammarids from other Aegean islands. More molecular data and thorough sampling of the area are essential to provide a detailed picture of the evolutionary history of Aegean freshwater insular gammarids. This is especially important given that Mediterranean islands are among the most anthropogenically affected regions in the Mediterranean region and are also one of the least studied in terms of their freshwater diversity^80^. Considering that the vast majority of freshwater ecosystems of the Aegean islands are under significant threat due to increasing water and habitat demands for tourism development, agriculture and pastoral development, there is an urgency for more studies revealing the actual biodiversity of insular freshwater biota that may aid in planning a reasonable and sustainable strategy for their conservation^80^. The results presented here provide not only additional evidence indicating strong connectivity between the evolution of the freshwater biota and the geological history of the Mediterranean, but also broaden the still scarce knowledge on the evolutionary mechanisms of the diversification of the insular freshwater fauna.

## Supplementary information

Supplementary Information 1.

Supplementary Information 2.

Supplementary Information 3.

Supplementary Information 4.

Supplementary Information 5.

Supplementary Information 6.

Supplementary Information 7.

Supplementary Information 8.

Supplementary Information 9.

## Data Availability

All DNA sequences and original chromatograms are stored in the BOLD database (10.5883/DS-GAEG), and the processed DNA sequences are also available in GenBank under following accession numbers (to be provided). The location data, respective alignments and additional metadata are also available from BOLD.
