# Supplementary material for: Molecular data suggest multiple origins and diversification times of freshwater gammarids on the Aegean archipelago
Source: Sci Rep. 2020 Nov 13;10:19813. doi: 10.1038/s41598-020-75802-2 (PMC7666221; doi:10.1038/s41598-020-75802-2)
Supplement: Supplementary file 1 — Supplementary Information 1. [file 41598_2020_75802_MOESM1_ESM.pdf]

Title: Molecular data suggest multiple origins and diversification times of freshwater gammarids on the Aegean Archipelago

Authors: Kamil Hupało, Ioannis Karaouzas, Tomasz Mamos, Michał Grabowski

Supplementary figures captions:

Fig.S1 Maximum clade credibility, time-calibrated Bayesian reconstruction of phylogeny of Aegean *Gammarus* species including the outgroups used for primary molecular clock calibration and validation. Phylogeny was inferred from sequences of the mitochondrial COI and 16S rRNA markersand nuclear 28S rRNA and EF1-alpha markers. The numbers by respective nodes indicate Bayesian posterior probability values ≥ 0.8. The coloured bars represent ABGD delimitation method, with colours correspond to those presented in other figures. Grey node bars represent 95% HPD. The calibrated node bars are highlighted, with 95% HPD of each calibrated node denoted in square brackets.

Fig.S2 Maximum clade credibility, time-calibrated Bayesian reconstruction of phylogeny of Aegean *Gammarus* species including the outgroups used for fossil molecular clock calibration and validation. Phylogeny was inferred from sequences of the mitochondrial COI and 16S rRNA genes and nuclear 28S rRNA gene and EF1-alpha gene. The numbers by respective nodes indicate Bayesian posterior probability values ≥ 0.8. The coloured bars represent ABGD delimitation method, with colours correspond to those presented in other figures. Grey node bars represent 95% HPD. The calibrated node bars are highlighted, with 95% HPD of each calibrated node denoted in square brackets.

Fig.S3 Lineage Through Time (LTT) plot for Aegean *Gammarus*. The black representing the median with grey lines representing 95% CI.

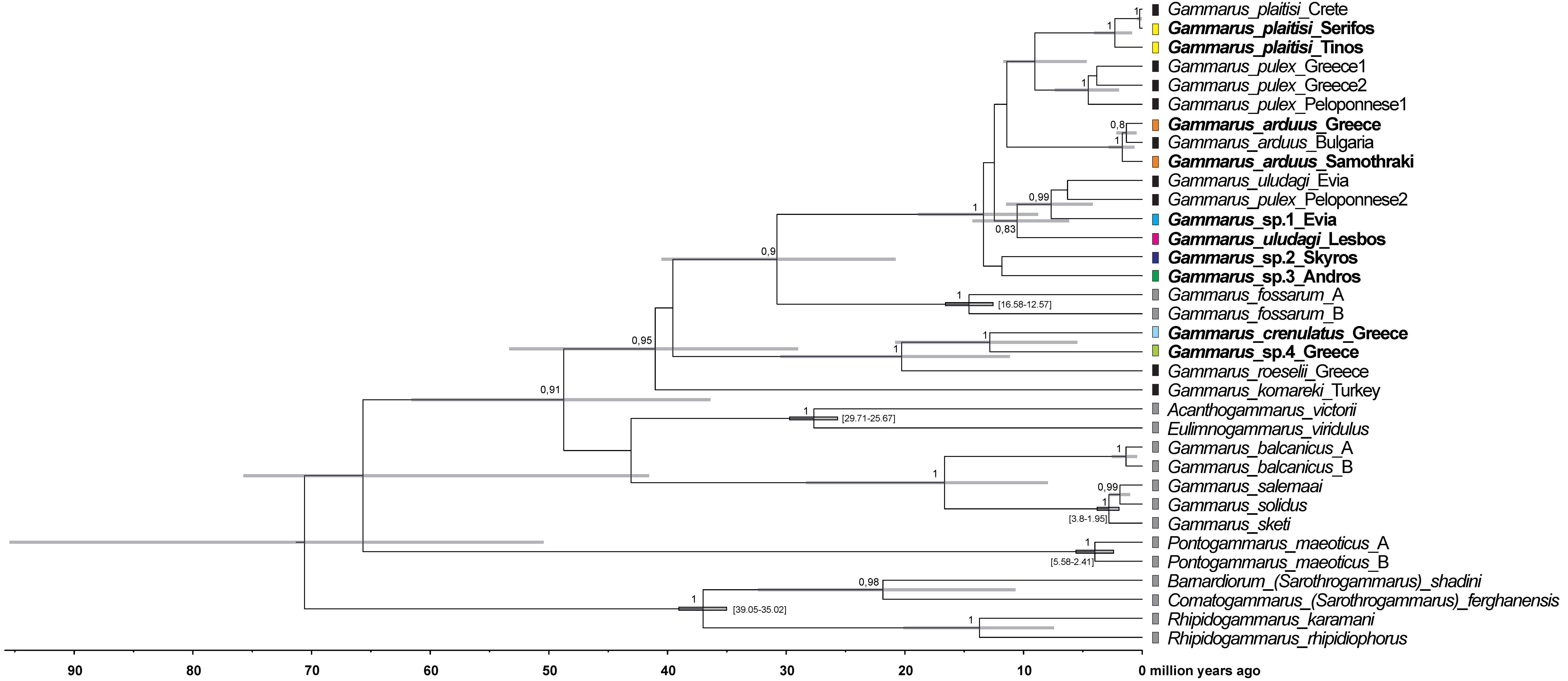

Gammarus_plaitisi_Crete
Gammarus_plaitisi_Serifos
Gammarus_plaitisi_Tinos
Gammarus_pulex_Greece1
Gammarus_pulex_Greece2
Gammarus_pulex_Peloponnese1
Gammarus_arduus_Greece
Gammarus_arduus_Bulgaria
Gammarus_arduus_Samothraki
Gammarus_uludagi_Evia
Gammarus_pulex_Peloponnese2
Gammarus_sp.1_Evia
Gammarus_uludagi_Lesbos
Gammarus_sp.2_Skyros
Gammarus_sp.3_Andros
Gammarus_fossarum_A
Gammarus_fossarum_B
Gammarus_crenulatus_Greece
Gammarus_sp.4_Greece
Gammarus_roeselii_Greece
Gammarus_komareki_Turkey
Acanthogammarus_victorii
Eulimnogammarus_viridulus
Gammarus_balcanicus_A
Gammarus_balcanicus_B
Gammarus_salemaai
Gammarus_solidus
Gammarus_sketi
Pontogammarus_maeoticus_A
Pontogammarus_maeoticus_B
Bamardiorum_(Sarothrogammarus)_shadini
Comatogammarus_(Sarothrogammarus)_ferghanensis
Rhipidogammarus_karamani
Rhipidogammarus_rhipidiophorus
[16.58-12.57]
[29.71-25.67]
[3.8-1.95]
[5.58-2.41]
[39.05-35.02]
0,8
0,99
0,83
0,9
0,95
0,91
0,98
90
80
70
60
50
40
30
20
10
0 million years ago

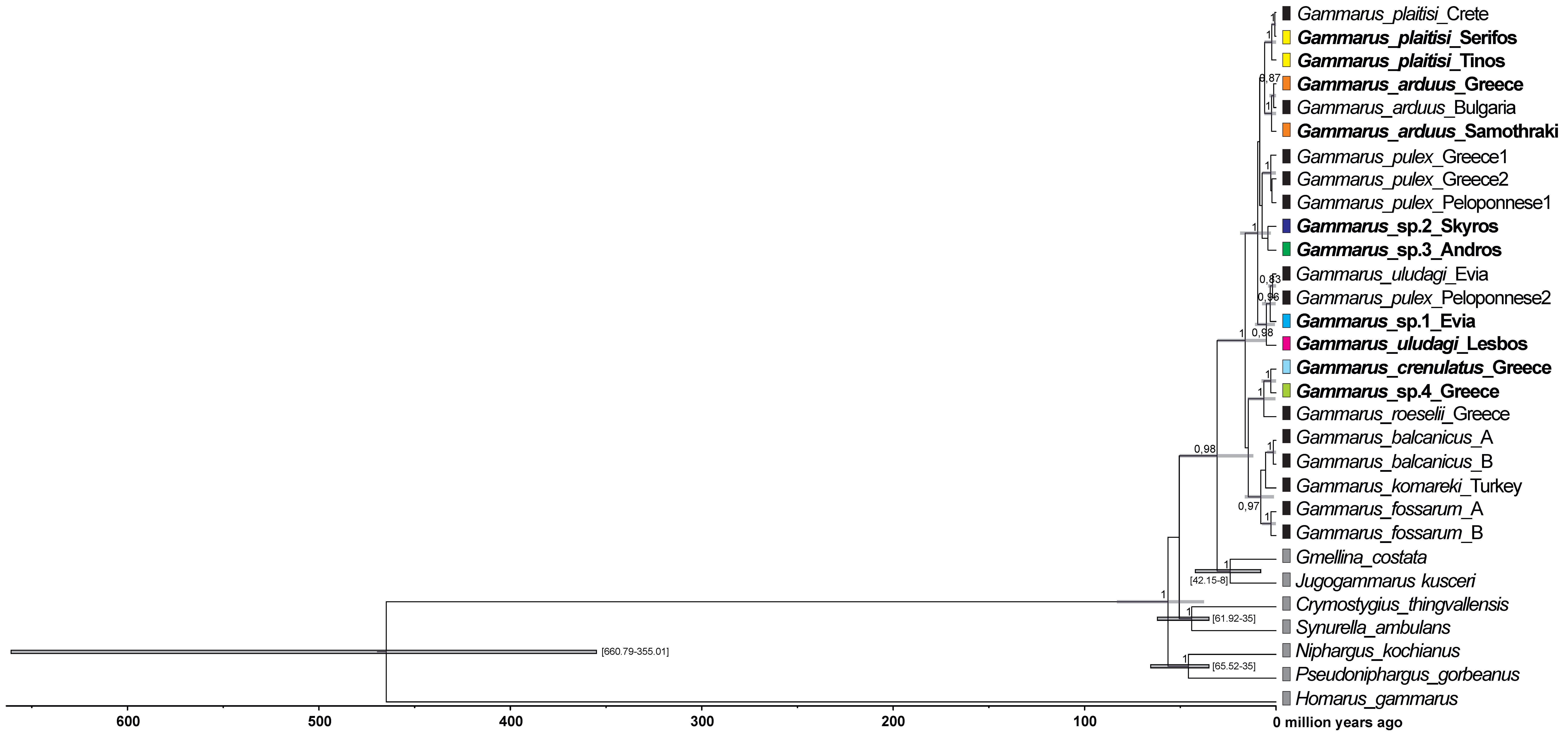

Gammarus_plaitisi_Crete
Gammarus_plaitisi_Serifos
Gammarus_plaitisi_Tinos
Gammarus_arduus_Greece
Gammarus_arduus_Bulgaria
Gammarus_arduus_Samothraki
Gammarus_pulex_Greece1
Gammarus_pulex_Greece2
Gammarus_pulex_Peloponnese1
Gammarus_sp.2_Skyros
Gammarus_sp.3_Andros
Gammarus_uludagi_Evia
Gammarus_pulex_Peloponnese2
Gammarus_sp.1_Evia
Gammarus_uludagi_Lesbos
Gammarus_crenulatus_Greece
Gammarus_sp.4_Greece
Gammarus_roeselii_Greece
Gammarus_balcanicus_A
Gammarus_balcanicus_B
Gammarus_komareki_Turkey
Gammarus_fossarum_A
Gammarus_fossarum_B
Gmellina_costata
Jugogammarus kusceri
Crymostygius_thingvallensis
Synurella_ambulans
Niphargus_kochianus
Pseudoniphargus_gorbeanus
Homarus_gammarus
0,87
0,83
0,96
0,98
0,98
0,97
[42.15-8]
[61.92-35]
[65.52-35]
[660.79-355.01]
600
500
400
300
200
100
0 million years ago

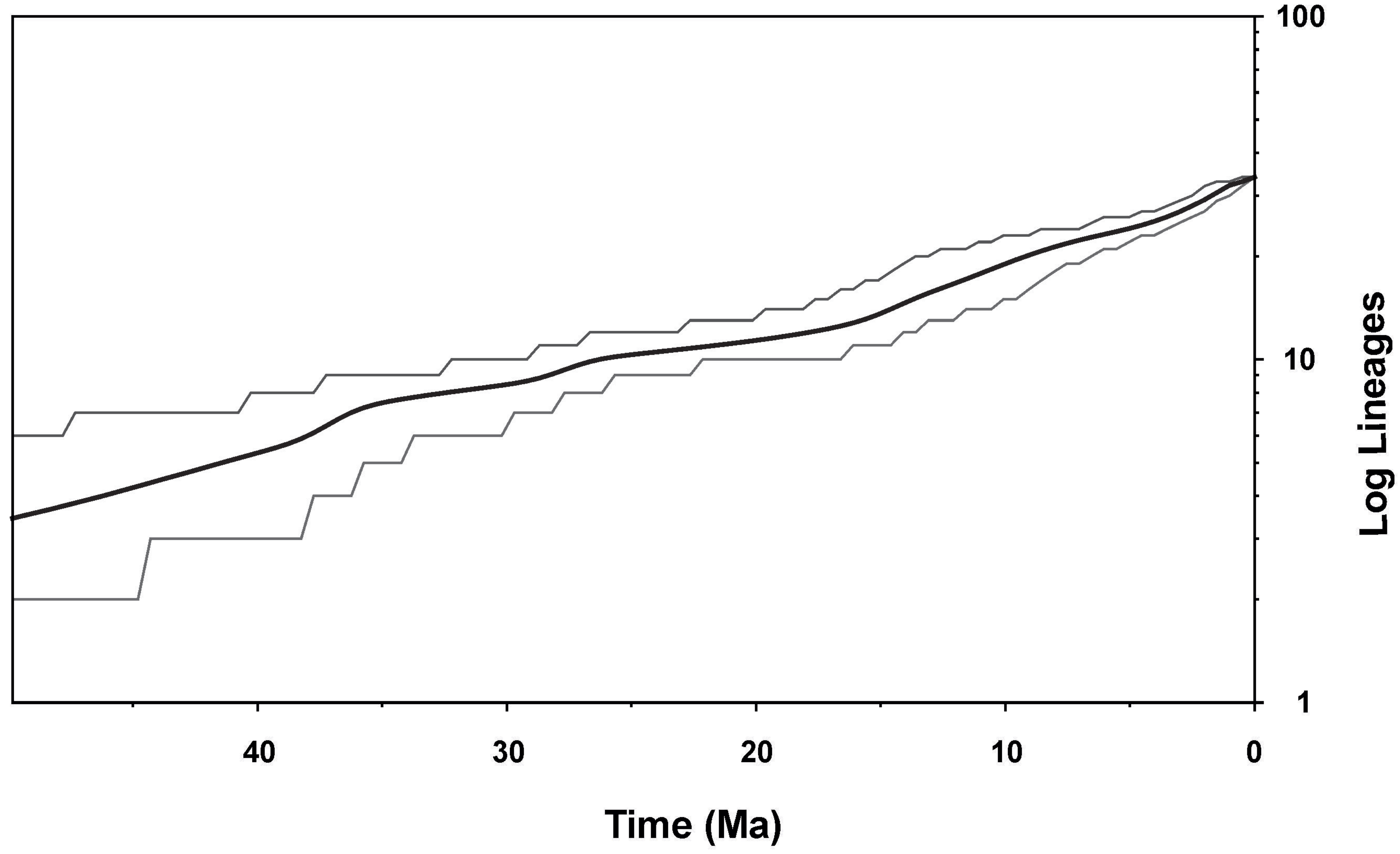
100
10
1
Log Lineages
40
30
20
10
0
Time (Ma)
