# Supplementary material for: Molecular data suggest multiple origins and diversification times of freshwater gammarids on the Aegean archipelago
Source: Sci Rep. 2020 Nov 13;10:19813. doi: 10.1038/s41598-020-75802-2 (PMC7666221; doi:10.1038/s41598-020-75802-2)
Supplement: Supplementary file 2 — Supplementary Information 2. [file 41598_2020_75802_MOESM2_ESM.docx]

Title: Molecular data suggest multiple origins and diversification times of freshwater gammarids on the Aegean Archipelago

Authors: Kamil Hupało, Ioannis Karaouzas, Tomasz Mamos, Michał Grabowski

Tab.S1 Used molecular markers with the primer sequences, PCR cycling conditions and the original references.

| Molecular marker | Primer sequences | PCR conditions | References |
| --- | --- | --- | --- |
| COI | LCO1490: 5'-ggtcaacaaatcataaagatattgg-3'  HC02198: 5'-taaacttcagggtgaccaaaaaatca-3' | 60 s at 94^o^C, 5x (30 s at 94 ^o^C, 90 s at 45^o^C, 60 s at 72^o^C), 35x (30 s at 94^o^C, 90 s at 51^o^C, 60 s at 72^o^C), 5 min at 72^o^C | Primer pairs: Folmer et al. 1994  PCR conditions: Hou et al. 2007 |
|  | LCO1490-JJ: 5'-chacwaaycataaagatatygg-3'  HCO2198-JJ: 5'-awacttcvggrtgvccaaaraatca-3' |  | Primer pairs: Astrin and Stüben, 2008  PCR conditions: Hou et al. 2007 |
| 16S rRNA | 16STf: 5'-ggtawhytracygtgctaag-3'  16SBr: 5’-ccggtttgaactcagatcatgt-3’ | 150 s at 94°C, 36x (40 s at 94°C, 40 s at 54°C, 80 s at 65°C), 8 min at 65°C | Primer pairs: Palumbi et al. 1991, MacDonald et al. 2005  PCR conditions: Weiss et al. 2014 |
| 28S rRNA | Niph15: 5’- caagtaccgtgagggaaagtt-3’  Niph16: 5’- agggaaacttcggagggaacc-3’ | 3min at 94°C, 50x (30 s at 94°C, 60 s at 45°C, 1min at 72°C), 5 min at 72°C | Primer pairs: Verovnik et al. 2005  PCR conditions: Verovnik et al. 2005 |
| EF-alpha | EF1aF: 5’- cactactggtcatctcatctac – 3’  EF1aR: 5’ – acttccaggagagtctcaaac – 3’ | 1min at 94°C, 35x (30 s at 94°C, 45 s at 50°C, 1min at 72°C), 5 min at 72°C | Primer pairs: Hou et al. 2011  PCR conditions: Hou et al. 2007 |
