# Supplementary material for: Molecular data suggest multiple origins and diversification times of freshwater gammarids on the Aegean archipelago
Source: Sci Rep. 2020 Nov 13;10:19813. doi: 10.1038/s41598-020-75802-2 (PMC7666221; doi:10.1038/s41598-020-75802-2)
Supplement: Supplementary file 3 — Supplementary Information 3. [file 41598_2020_75802_MOESM3_ESM.docx]

Title: Molecular data suggest multiple origins and diversification times of freshwater gammarids on the Aegean Archipelago

Authors: Kamil Hupało, Ioannis Karaouzas, Tomasz Mamos, Michał Grabowski

Tab.S2 Reference sequences (mined from GenBank) used in the reconstruction of the phylogeny in this study.

| **Species** | **Locality  (Aegean Island/Country)** | **GenBank Accession Numbers** | **Reference study** |
| --- | --- | --- | --- |
| *Gammarus alpinus* | Switzerland | COI: KX283242 | Alther et al. (2016) |
| *Gammarus arduus* | Bulgaria | COI: MH493848; 16S: MH496581; EF-α: MH493860 | Copilaș‐Ciocianu et al. (2018) |
| *Gammarus balcanicus* | Bulgaria | COI: KU056256; 16S: KU056052; 28S: KU056154 | Mamos et al. (2016) |
|  | Romania | COI: KU056394; 16S: KU056103; 28S: KU056154 | Mamos et al. (2016) |
| *Gammarus crenulatus* | Greece | COI: KJ462752 | Wysocka et al. (2014) |
| *Gammarus fossarum* | Romania | COI: KR061783; 16S: KR061718; 28S: KR061765 | Copilaş-Ciocianu & Petrusek, (2015) |
|  | Albania | COI: KJ462736 | Wysocka et al. (2014) |
|  | Bulgaria (1) | COI: KJ462737 | Wysocka et al. (2014) |
|  | Bulgaria (2) | COI: KJ462738 | Wysocka et al. (2014) |
| *Gammarus komareki* | Turkey | COI: KJ462758; 16S: KJ462574; 28S: KJ462656 | Wysocka et al. (2014) |
|  | Greece | COI: KJ462739 | Wysocka et al. (2014) |
| *Gammarus lacustris* | Finland | COI: KX283246 | Alther et al. (2016) |
| *Gammarus plaitisi* | Crete (A); Spring in Sfinari beach | COI: MG784518 | Hupało et al. (2018) |
|  | Crete (B); Pelekaniotikos river | COI: MG784477 | Hupało et al. (2018) |
|  | Crete; Fodele, locus typicus | COI: MG784515; 16S: MG784372 ; 28S: MG784428; EF-α: MG792358 | Hupało et al. (2018) |
| *Gammarus pulex* | Greece (1) | COI: KJ462741, 16S: KJ462557; 28S: KJ462639 | Wysocka et al. (2014) |
|  | Greece (2) | COI: KJ462768; 16S: KJ462584; 28S: KJ462685 | Wysocka et al. (2014) |
|  | Peloponnese (1) | COI: MG784489; 16S: MG784379; 28S: MG784423; EF-α: MG792353 | Hupało et al. (2018) |
|  | Peloponnese (2) | COI: MG784478,MG784486,MG784481,MG784485; 16S: MG784354; 28S: MG784426; EF-α: MG792354 | Hupało et al. (2018) |
|  | Sweden | COI: JF965943,JF965939 | Hou et al. (2011) |
| *Gammarus roeselii* | Greece | COI: JF965983; 28S: JF965817 | Hou et al. (2011) |
| *Gammarus rambouseki* | Albania (1) | COI: KJ462742 | Wysocka et al. (2014) |
|  | Albania (2) | COI: KJ462744 | Wysocka et al. (2014) |
|  | Albania (3) | COI: KJ462746 | Wysocka et al. (2014) |
| *Gammarus uludagi* | Evia | COI: JF965986; 28S: JF965817; EF-α: JF966112 | Hou et al. (2011) |
|  | Albania | COI: KJ462750 | Wysocka et al. (2014) |
| *Asellus aquaticus* | Ukraine | COI: KR921859 | Sworobowicz et al. (2015) |
