# Supplementary material for: Molecular data suggest multiple origins and diversification times of freshwater gammarids on the Aegean archipelago
Source: Sci Rep. 2020 Nov 13;10:19813. doi: 10.1038/s41598-020-75802-2 (PMC7666221; doi:10.1038/s41598-020-75802-2)
Supplement: Supplementary file 4 — Supplementary Information 4. [file 41598_2020_75802_MOESM4_ESM.docx]

Title: Molecular data suggest multiple origins and diversification times of freshwater gammarids on the Aegean Archipelago

Authors: Kamil Hupało, Ioannis Karaouzas, Tomasz Mamos, Michał Grabowski

Tab.S3 Results of saturation tests.

| **Molecular marker** | **Iss value** | **Iss.c value** | **P value** |
| --- | --- | --- | --- |
| **COI** | 0,616 | 0,777 | 0,0038 |
| **16S** | 0,5134 | 0,5375 | 0,0088 |
| **28S** | 0,2187 | 0,7016 | <0.0001 |
| **EF-alpha** | 0,0519 | 0,6962 | <0.0001 |
