# Supplementary material for: Molecular data suggest multiple origins and diversification times of freshwater gammarids on the Aegean archipelago
Source: Sci Rep. 2020 Nov 13;10:19813. doi: 10.1038/s41598-020-75802-2 (PMC7666221; doi:10.1038/s41598-020-75802-2)
Supplement: Supplementary file 5 — Supplementary Information 5. [file 41598_2020_75802_MOESM5_ESM.docx]

Title: Molecular data suggest multiple origins and diversification times of freshwater gammarids on the Aegean Archipelago

Authors: Kamil Hupało, Ioannis Karaouzas, Tomasz Mamos, Michał Grabowski

Tab.S4 The best-fit substitution models for used molecular markers determined by bModel test.

| **Molecular marker** | **Codon positions** | **Model number** | **Likelihood percentage** | **Substitution model** |
| --- | --- | --- | --- | --- |
| **COI** | 1 | 123141 | 44,91% | TN93 + G + I |
|  | 2 | 123423 | 24,21% | TIM + G + I |
|  | 3 | 121131 | 50,43% | TN93 + G + I |
| **16S** | 1,2,3 | 123123 | 20,81% | TN93 + G + I |
| **28S** | 1,2,3 | 123453 | 18,42% | TIM + G + I |
| **EF alpha** | 1,2 | 121123 | 9,05% | HKY + G + I |
|  | 3 | 121323 | 25,17% |  |
|  |  |  |  | TN93 + G + I |
|  |  |  |  |  |
