# Supplementary material for: Molecular data suggest multiple origins and diversification times of freshwater gammarids on the Aegean archipelago
Source: Sci Rep. 2020 Nov 13;10:19813. doi: 10.1038/s41598-020-75802-2 (PMC7666221; doi:10.1038/s41598-020-75802-2)
Supplement: Supplementary file 6 — Supplementary Information 6. [file 41598_2020_75802_MOESM6_ESM.docx]

Title: Molecular data suggest multiple origins and diversification times of freshwater gammarids on the Aegean Archipelago

Authors: Kamil Hupało, Ioannis Karaouzas, Tomasz Mamos, Michał Grabowski

Tab.S5 Reference sequences used as primary calibration points for the calibration of the molecular clock for the reconstruction of the time-calibrated phylogeny.

| **Species** | **Calibration time** | **Calibration priors** | **GenBank Accession numbers** | **Reference study** |
| --- | --- | --- | --- | --- |
| *Gammarus salemaai* | 2 ± 1 Ma  (f. *G. sketi*) | Log Normal  M: 0.7  S: 0.2 | COI: JX899266 | Wysocka et al. (2013) |
|  |  |  | 16S: JX899088 |  |
|  |  |  | 28S: KJ462668 | Wysocka et al. (2014) |
| *Gammarus sketi* | 2 ± 1 Ma  (f. *G. solidus,G. salemai*) |  | COI: JX899272 | Wysocka et al. (2013) |
|  |  |  | 16S: JX899094 |  |
|  |  |  | 28S: KJ462672 | Wysocka et al. (2014) |
| *Gammarus solidus* | 2 ± 1 Ma  (f. *G. sketi*) |  | COI: JX899282 | Wysocka et al., (2013) |
|  |  |  | 16S: JX899104 |  |
|  |  |  | 28S: KJ462673 | Wysocka et al. (2014) |
| *Pontogammarus maeoticus* (Black Sea) | 4 ± 2 Ma  (f. *P. maeoticus* Caspian) | Log Normal  M: 1.386  S: 0.25 | COI: AY189494 | Cristescu et al. (2003) |
|  |  |  | 28S: AY529062 | Cristescu & Hebert, (2005) |
| *Pontogammarus maeoticus* (Caspian Sea) | 4 ± 2 Ma  (f. *P. maeoticus* Black) |  | COI: AY189500 | Cristescu et al. (2003) |
|  |  |  | 28S: AY529063 | Cristescu & Hebert, (2005) |
| *Gammarus fossarum* G | 15 ± 2 Ma  (f. *G. fossarum* M) | Log Normal  M: 2.71  S: 0.07 | COI: KR061783 | Copilaş-Ciocianu & Petrusek, (2015) |
|  |  |  | 16S: KR061718 |  |
|  |  |  | 28S: KR061765 |  |
| *Gammarus fossarum* M | 15 ± 2 Ma  (f. *G. fossarum* G) |  | COI: KR061823 | Copilaş-Ciocianu & Petrusek, (2015) |
|  |  |  | 16S: KR061694 |  |
|  |  |  | 28S: KR061753 |  |
| *Acanthogammarus victorii* | 28 ± 2 Ma  (f. *E. viridulus*) | Log Normal  M: 3.333  S: 0.038 | COI: AY926652 | MacDonald et al. (2005) |
|  |  |  | 16S: AY926695 |  |
| *Eulimnogammarus viridulus* | 28 ± 2 Ma  (f. *A. victorii*) |  | COI: AY926665 | MacDonald et al. (2005) |
|  |  |  | 16S: AY926715 |  |
| *Sarothrogammarus (Comatogammarus) ferghanensis* | 37 ± 2 Ma (f. *Rhipidogammarus*) | Log Normal  M: 3.61  S: 0.028 | COI: JF965996 | Hou et al. (2011) |
|  |  |  | 28S: JF965828 |  |
| *Sarothrogammarus (Barnardiorum) shadini* | 37 ± 2 Ma (f. *Rhipidogammarus*) |  | COI: JF965994 | Hou et al. (2011) |
|  |  |  | 28S: JF965826 |  |
| *Rhipidogammarus karamani* | 37 ± 2 Ma (f. *Sarothrogammarus*) |  | COI: JF965993 | Hou et al. (2011) |
|  |  |  | 28S: JF965825 |  |
| *Rhipidogammarus rhipidiophorus* | 37 ± 2 Ma (f. *Sarothrogammarus*) |  | COI: JF965992 | Hou et al. (2011) |
|  |  |  | 28S: JF965824 |  |
|  |  |  | 16S: AY926715 |  |
