# Supplementary material for: Molecular data suggest multiple origins and diversification times of freshwater gammarids on the Aegean archipelago
Source: Sci Rep. 2020 Nov 13;10:19813. doi: 10.1038/s41598-020-75802-2 (PMC7666221; doi:10.1038/s41598-020-75802-2)
Supplement: Supplementary file 7 — Supplementary Information 7. [file 41598_2020_75802_MOESM7_ESM.docx]

Title: Molecular data suggest multiple origins and diversification times of freshwater gammarids on the Aegean Archipelago

Authors: Kamil Hupało, Ioannis Karaouzas, Tomasz Mamos, Michał Grabowski

Tab.S6 Reference sequences used as fossil calibration points for the calibration of the molecular clock for the reconstruction of the time-calibrated phylogeny.

| **Fossil calibration point** | **Species** | **Calibration time** | **Calibration priors** | **GenBank Accession numbers** | **Reference study** |
| --- | --- | --- | --- | --- | --- |
| **1** | *Synurella ambulans* | 126.5 ± 88.5 Ma  (f. *C. thingvallensis* ) | Exponential  Mean: 60.0  Offset: 35.0 | COI: KF290224 | Flot et al. (2014) |
|  |  |  |  | 16S: HQ286000 | Kornobis et al. (2011) |
|  |  |  |  | 28S: KF290032 | Flot et al. (2014) |
|  | *Crymostygius thingvallensis* | 126.5 ± 88.5 Ma  (f. *S. ambulans*) |  | COI: HQ286032 | Kornobis et al. (2011) |
|  |  |  |  | 16S: HQ286009 |  |
|  |  |  |  | 28S: HQ286019 |  |
| **2** | *Niphargus kochianus* | 126.5 ± 88.5 Ma  (f. *P. gorbeanus*) | Exponential  Mean: 60.0  Offset: 35.0 | COI: KC315668 | McInerney et al. (2014) |
|  |  |  |  | 16S: KC315567 |  |
|  |  |  |  | 28S: EU693308 | Fišer et al. (2008) |
|  | *Pseudoniphargus gorbeanus* | 126.5 ± 88.5 Ma  (f. *N. kochianus*) |  | COI: KY441038 | Jurado-Rivera et al. (2017) |
|  |  |  |  | 28S: KY441101 |  |
| **3** | *Gmelina costata* | 46 ± 37 Ma  (f. *J. kusceri*) | Exponential  Mean: 25.0  Offset: 8.0 | 28S: KF478468 | Hou et al. (2014) |
|  | *Jugogammarus kusceri* | 46 ± 37 Ma  (f. *G. aestuarica*) |  | COI: KF478552 | Hou et al. (2014) |
|  |  |  |  | 28S: KF478462 |  |
| **4** | *Homarus gammarus* | 436 ± 78 Ma  (f. all other taxa in this study) | Exponential  Mean: 55.0  Offset: 355.0 | COI: KT209166 | Raupach et al. (2015) |
|  |  |  |  | 16S: DQ079714 | Porter et al. (2015) |
|  |  |  |  | 28S: DQ079789 |  |
