# Supplementary material for: Molecular data suggest multiple origins and diversification times of freshwater gammarids on the Aegean archipelago
Source: Sci Rep. 2020 Nov 13;10:19813. doi: 10.1038/s41598-020-75802-2 (PMC7666221; doi:10.1038/s41598-020-75802-2)
Supplement: Supplementary file 8 — Supplementary Information 8. [file 41598_2020_75802_MOESM8_ESM.docx]

Title: Molecular data suggest multiple origins and diversification times of freshwater gammarids on the Aegean Archipelago

Authors: Kamil Hupało, Ioannis Karaouzas, Tomasz Mamos, Michał Grabowski

Tab.S7 Cross-validation of the ages of the nodes obtained by using primary calibration points with the dating obtained through fossil calibration.

| **Species** | **Node description** | **Molecular dating range 95% HPD**  **(primary calibration)** | **Molecular dating range 95% HPD**  **(fossil calibration)** |
| --- | --- | --- | --- |
| ***Gammarus plaitisi*** | Divergence between Tinos and Serifos/Crete populations | 0.85 – 4.05 Ma | 0.04 – 5.96 Ma |
| ***Gammarus arduus*** | Divergence between the population from Samothraki with the mainland conspecifics | 0.65 – 2.82 Ma | 0.06 – 6.04 Ma |
| ***Gammarus uludagi/sp.1*** | Divergence between *G. uludagi* from Lesbos with *G*. sp1 and *G. uludagi* from Evia | 6.16 – 14.3 Ma | 0.57 – 10.93 Ma |
| ***Gammarus sp.2/sp.3*** | Divergence between *G*. sp.2 from Skyros from *G*. sp.3 from Andros | 4.8 – 15.68 Ma | 2.68 – 17.81 Ma |
| ***Gammarus sp.4/crenulatus*** | Divergence of both *G.* sp.4 and *G. crenulatus* from outgroup *G. roeselii* | 11.14 – 30.69 Ma | 0.3 – 14.69 Ma |
