# Supplementary material for: Molecular data suggest multiple origins and diversification times of freshwater gammarids on the Aegean archipelago
Source: Sci Rep. 2020 Nov 13;10:19813. doi: 10.1038/s41598-020-75802-2 (PMC7666221; doi:10.1038/s41598-020-75802-2)
Supplement: Supplementary file 9 — Supplementary Information 9. [file 41598_2020_75802_MOESM9_ESM.docx]

Title: Molecular data suggest multiple origins and diversification times of freshwater gammarids on the Aegean Archipelago

Authors: Kamil Hupało, Ioannis Karaouzas, Tomasz Mamos, Michał Grabowski

Tab.S8 Comparison of mean values of the genetic distance calculation methods.

| **MOTU** | **Genetic distance  to the closest MOTU (Pat/K2p)** |
| --- | --- |
| **MOTU1**  *(Gammarus plaitisi)* | Patristic **17,6%/17,3%** K2p |
| **MOTU2**  *(Gammarus arduus)* | Patristic **17,2%/17%** K2p |
| **MOTU3**  *(Gammarus sp.1)* | Patristic **14,2%/14%** K2p |
| **MOTU4**  *(Gammarus* sp.*2)* | Patristic **16,5%/16,7%** K2p |
| **MOTU5**  *(Gammarus* sp.*3)* | Patristic **18%/18,1%** K2p |
| **MOTU6** *(Gammarus uludagi)* | Patristic **18%/18,1%** K2p |
| **MOTU7**  *(Gammarus* *birsteini)* | Patristic **18,8%/20,5%** K2p |
| **MOTU8** *(Gammarus crenulatus)* | Patristic **18,8%/20,5%** K2p |
